# AAV-mediated gene transfer of human pigment epithelium-derived factor inhibits Lewis lung carcinoma growth in mice

**DOI:** 10.3892/or.2012.1621

**Published:** 2012-01-04

**Authors:** SHA-SHA HE, HUA-SHAN SHI, TAO YIN, YONG-XIA LI, SHUN-TAO LUO, QIN-JIE WU, LIAN LU, YU-QUAN WEI, LI YANG

**Affiliations:** State Key Laboratory of Biotherapy, West China Hospital, Sichuan University, Chengdu 610041, P.R. China

**Keywords:** AAV-PEDF, LLC, tumour, apoptosis, angiogenesis

## Abstract

Pigment epithelium-derived factor (PEDF) is the most potent inhibitor of angiogenesis in the mammalian eye, and mechanisms through which PEDF exerts its antitumour activity have recently been defined. The aim of our research was to evaluate the ability of adeno-associated virus (AAV) vector-mediated transfer of human PEDF to inhibit Lewis lung carcinoma (LCC) cell growth. Intratumoural injection of AAV-PEDF caused significant reduction of the tumour volume and prolonged the survival time of mice bearing LLC cells, which were associated with decreased microvessel density and increased apoptosis in the tumours. AAV vectors represent a very promising tool for cancer gene therapy. No noticeable toxicity concerning AAV was detected as inferred from monitoring changes in animal body weight as well as basic organ structure and histological morphology, and by analyzing mouse liver and kidney function. Our findings indicate that AAV-mediated PEDF gene expression may offer an active approach to inhibit LLC growth and that treatment with AAV-PEDF may provide a promising therapeutic strategy in lung cancer treatment.

## Introduction

Extensive research has established that neoangiogenesis plays a pivotal role in solid malignant tumour growth and metastasis (1–3). Tumours cannot exceed a few millimetres in diameter without the development of a neovasculature supply. Thus, antiangiogenesis therapy is a potentially promising tumouristatic approach (4–6). However, evidence has emerged that angiogenesis is tightly regulated by a balance of activating and inhibiting factors (7,8). Therefore, long-term overexpression of angiogenesis suppressors may be required for effectively controlling tumour proliferation through counteracting tumour-induced angiogenesis.

Pigment epithelium-derived factor (PEDF) is a 50-kDa secreted glycoprotein that belongs to the serine protease inhibitor superfamily but lacks protease inhibitor activity (9–11). PEDF was first identified and purified from the conditioned medium of cultured human neonatal retinal pigment epithelial cells (9,12). This factor is involved in multiple and varied biological activities (13), which makes it an appealing potential treatment for Lewis lung carcinoma (LCC). With regard to its antiangiogenic activity, PEDF is more potent than any other endogenous inhibitors of neovascularisation (14,15); this property makes PEDF an excellent candidate for LLC treatment as a form of targeted gene therapy. The antiangiogenic potency of PEDF has been shown to inhibit tumour angiogenesis in several preclinical cancer models (16–27). In addition, PEDF is thought to exert its antiangiogenic activity through two major pathways, namely, endothelial cell apoptosis via activation of the Fas/Fas-L death pathway (28), and disruption of the crucial balance between pro- and anti-angiogenic factors, via downregulation of vascular endothelial growth factor (VEGF) expression (29–31). Furthermore, a few recent reports indicate that PEDF not only acts to halt angiogenesis, but also has the ability to increase apoptosis in tumours (16–18,26). This apoptotic activity is likely due to a distinct functional epitope on the PEDF protein (20).

Successful antiangiogenic therapy requires efficient and continuous secretion of the candidate protein for long periods of time. Gene transfer is usually utilized as an effective strategy for chronic delivery of antiangiogenic factors. Adeno-associated virus (AAV) vectors represent a very promising tool for cancer gene therapy because they are capable of sustained, long-term gene expression, non-pathogenicity, low immunogenicity, and they lack cytotoxicity (32–34).

In this study, we constructed AAV vectors that express PEDF in order to investigate the effect of AAV-mediated intratumoural PEDF expression on LLC tumour suppression.

## Materials and methods

### Cell lines and culture

HUVECs were isolated and cultured in DMEM medium (Gibco-BRL, NY, USA) supplemented with 20% FBS and 100 g/ml bovine fibroblast growth factor (BFGF). LLC cells were obtained from the American Type Culture Collection (ATCC, Rockville, MD, USA) and cultured in DMEM medium (Gibco-BRL) supplemented with 10% FBS and 100 μg/ml amikacin.

### AAV-PEDF preparation

AAV-PEDF was constructed using CMV as the promoter. cDNAs containing full-length human PEDF sequences under the CMV promoter were cloned. The construct sequence was confirmed via DNA sequencing (Invitrogen Inc., Shanghai). A control virus containing a green fluorescent protein cDNA under the same promoter (AAV-EGFP) was also detected by DNA sequencing (Invitrogen Inc.). Packaging and purification of rAAV particles were performed as previously described (35).

### Cell infection with AAV-PEDF

LLC cells were seeded in 6-well plates. After an overnight culture, the cells were infected with AAV-PEDF and AAV-EGFP viruses at a multiplicity of infection (MOI) of 1×10^5^ infectious particles/cell. The cells and supernatants were collected after 72 h.

### Western blot analysis

LLC cells seeded in the 6-well plates were harvested and resuspended in lysis buffer while the LLC tumours were grinded and then lysed with RIPA solution, respectively. Equal amounts of protein were separated by SDS-polyacrylamide gel electrophoresis (PAGE) and then electrotransferred onto a polyvinylidene difluoride membrane (PVDF). Blots were probed with a goat anti-human PEDF monoclonal antibody (1:1000, mAb; R&D Systems, Boston, MA, USA) plus a secondary biotinylated antibody against goat IgG (1:10,000, ZSGB-BIO, Beijing, China). Immunoreactivity was detected using an enhanced chemiluminescence (ECL) detection system (Pierce, Rockford, IL, USA).

### Animal experiments

Male C57BL/6 mice were purchased from the Experimental Animal Centre at Sichuan University. All animal experimental procedures were approved by the West China Hospital Cancer Centre’s Animal Care and Use Committee. Aliquots of LLC cells (5×10^5^) were subcutaneously inoculated into the mice. When the average tumour volume reached 90–100 mm^3^ in size, the mice were randomly divided into 3 groups. Each mouse in the AAV-PEDF group was treated with an intratumoural injection of 2×10^10^ AAV-PEDF virus particles. The mice in the control groups received 2×10^10^ AAV-EGFP particles or normal saline (NS). Tumour sizes and animal weights were measured every three days. The tumour volumes (mm^3^) were calculated according to the following formula: (length × width^2^ × 0.52). Mouse sera were collected for liver and kidney function analyses on the fifteenth day post-treatment using an AU7020 Automatic Biochemical Analyzer (Hitachi).

### TUNEL assay, immunohistochemistry analysis and H&E staining

Apoptotic tumour cells were determined by the DeadEnd colorimetric terminal deoxynucleotidyl transferase-mediated dUTP nick-end labelling (TUNEL) System (Promega Corp., Madison, USA) and the caspase-3 immunohistochemical assay. Prepared tumour cryosections were incubated with primary anti-human PEDF antibody (1:200, mAb; R&D Systems) overnight, then with biotinylated anti-goat IgG secondary antibody (ZSJQ Biotechnology) and finally with diaminobenzidine (DAB; ZSJQ Biotechnology) as a substrate for visualization of the antigen-antibody complex. Frozen tumour specimens were analyzed by CD31 immunohistochemistry. The microvessel density was quantified using the reported method of Weidner *et al* (36). Paraffin sections were also stained with haematoxylin and eosin (H&E) to observe the structure and histological morphology of the tumours and basic organs.

### Alginate-encapsulated tumour cell and tube formation assay

LLC cells were resuspended in alginate solution. LLC cells in alginate solution were dropped into a swirling 0.25 M CaCl_2_ solution to prepare alginate beads (1×10^5^ cells/bead). Male C57Bl/6 mice were implanted s.c. with alginate beads into the back (1 bead/side). The next day, the mice were treated with i.v. administration of 2×10^10^ particles AAV-PEDF per mouse, or 2×10^10^ particles AAV-EGFP per mouse or with NS.

On Day 11 after treatment, 0.1 ml of 1% FITC-dextran solution (100 mg/kg) was injected i.v. into the tail vein of the mice. Alginate beads were photographed and rapidly removed 20 min after FITC-dextran injection. The beads were then vortexed and centrifuged in tubes containing 2 ml NS and the supernatant fluorescence was measured.

The tube formation assay was performed using Matrigel (BD Biosciences, San Jose, CA) that was thawed overnight at 4°C. Pre-chilled 24-well plates were coated with 300 μl/well of Matrigel (BD Biosciences). HUVECs were seeded in each well at a concentration of 1×10^5^ cells, and then treated with conditioned medium (CM) from LLC cells infected *in vitro* with AAV-PEDF, AAV-EGFP or NS. Tubule branches were photographed 6 h after incubation.

### Statistical analysis

Values are presented as means ± SD. Statistical analysis was conducted using the SPSS program (version 18.0, SPSS Inc., Chicago, IL, USA). The statistical significances were calculated by one-way ANOVA. The Kaplan-Meier method was used to evaluate survival curves and survival rate among groups. A P-value <0.05 was considered significant.

## Results

### PEDF gene expression in vivo and in vitro

To confirm PEDF gene expression *in vivo* and *in vitro*, western blotting was performed. PEDF protein was detected only in LLC tumours from AAV-PEDF treated mice (Fig. 2B). Human PEDF was detected as a single 50 kDa band in AAV-PEDF transduced cells (Fig. 2C). No PEDF was found in the lysates from AAV-EGFP-transduced or NS-treated cells. Seven days after inoculation, tumours were injected with AAV-EGFP, AAV-PEDF, or NS, and only the cytoplasm of LLC cells treated with AAV-PEDF clearly was stained for intratumoural PEDF (Fig. 2A). These results demonstrate successful PEDF gene expression *in vivo* and *in vitro*.

### Intratumoural treatment of AAV-PEDF suppresses tumour growth and prolongs mouse survival rate

**Figure 1:**
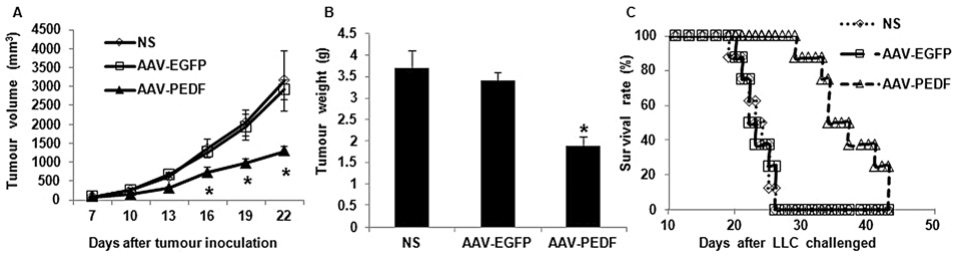
Antitumour efficacy of AAV-PEDF *in vivo*. (A) LLC cells were inoculated into C57BL/6 mice. When the average tumour size reached 90–100 mm^3^, the mice received an intratumoural injection of AAV-PEDF, AAV-EGFP or normal saline (NS). Tumour volumes were measured and calculated. (B) Mice were sacrificed on Day 15 after LLC inoculation and tumours were weighed. (C) Survival curves of mice. Significant increases in survival rates and prolonged survival times were observed in AAV-PEDF-treated mice. N=8 per group. ^*^P<0.05 compared to AAV-EGFP or the NS control groups.

**Figure 2:**
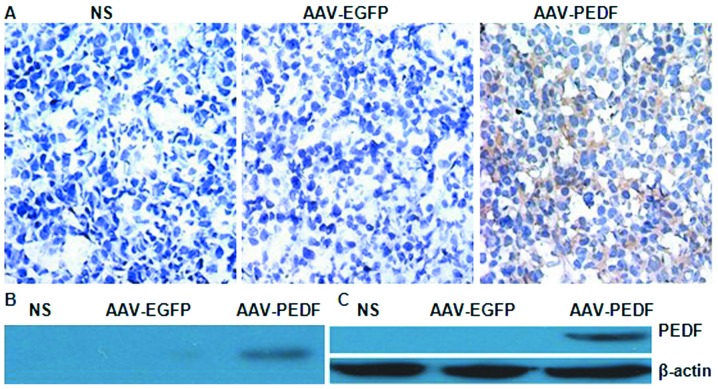
PEDF gene expression *in vivo* and *in vitro*. Mice were euthanized on day 15 post-treatment and (A) tumours were processed for immunohistochemistry analysis (original magnification, ×400). Only the cytoplasm of LLC cells treated with AAV-PEDF was stained for intratumoural PEDF. (B) PEDF protein (50 kDa) was detected in AAV-PEDF, but not AAV-EGFP or normal-saline (NS)-treated LLC tumours. (C) Human PEDF was detected as a single band of 50 kDa in AAV-PEDF-infected cells, but not in AAV-EGFP-infected or NS-treated cells.

Established subcutaneous LLC tumours in the C57BL/6 mouse model were used to investigate the antitumoural efficacy of AAV-PEDF by intratumoural injection. There were no significant differences in size between those tumours receiving NS vs. AAV-EGFP (Fig. 1A). In contrast, the growth of AAV-PEDF-treated LLC tumours was significantly inhibited compared with either AAV-EGFP or NS (Fig. 1A, P<0.05). The maximum tumour growth inhibition was observed on day 15 after treatment (56 and 58% inhibition, respectively, compared with mice treated with AAV-EGFP or NS, Fig. 1A). Tumour weights in the AAV-PEDF group were significantly lower than either the AAV-EGFP or NS groups (Fig. 1B, P<0.05). In addition, intratumoural treatment of AAV-PEDF resulted in prolonged animal survival (Fig. 1C, P<0.05).

### AAV-PEDF treatment decreases microvessel density and increases apoptosis in tumour tissue

**Figure 3:**
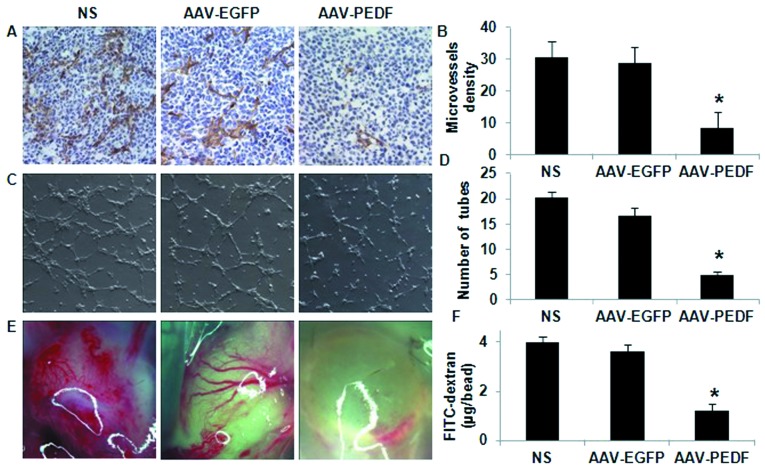
CD31 immunohistochemistry, tube formation, and Alginate-encapsulated tumour cell assay. (A) Micrographs show tumour tissue sections stained with anti-CD31 antibody (original magnification, ×400) after NS, AAV-EGFP, or AAV-PEDF treatment. (B) Quantitative results of microvessel density (MVD) in tumour tissue. The AAV-PEDF group showed a significant decrease in MVD compared to control groups (^*^P<0.05). Endothelial cells form tube and capillary-like structures on a Matrigel membrane through a process involving attachment, alignment and migration. (C) Treatment with conditioned medium (CM) from LLC cells infected *in vitro* with AAV-PEDF significantly decreased this process (original magnification, ×100). (D) The tube formations were quantified by counting the number of connecting branches between 2 discrete endothelial cells (^*^P<0.05). (E) Photographs show alginate bead surfaces from different groups. (F) FITC-dextran uptake in the tumour was significantly decreased in mice treated with AAV-PEDF compared to the control groups (^*^P<0.05).

Tumours from AAV-PEDF-treated animals showed marked reduction in microvessel density (MVD) (Fig. 3A). Quantitative analysis showed a 71 and 73% reduction in intratumoural MVD in AAV-PEDF treated animals compared with control animals receiving AAV-EGFP and NS, respectively (Fig. 3B). There was no significant difference in vessel density between tumours that received NS or AAV-EGFP (Fig. 3B; NS vs. AAV-EGFP, P>0.1).

**Figure 4:**
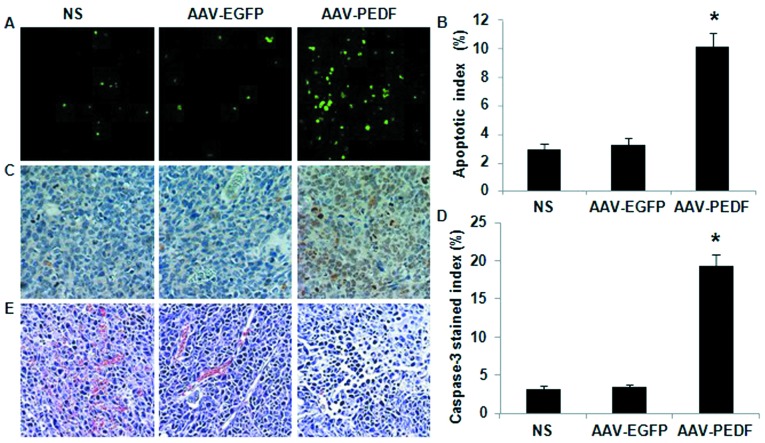
TUNEL, caspase-3 immunohistochemical assay, and histological staining for tumour tissue. (A) Apoptotic cells within LLC tumours were detected by the TUNEL assay (original magnification, ×400). (B) The apoptotic index (%) of LLC tumours treated with NS, AAV-EGFP, and AAV-PEDF (^*^P<0.05). (C) Micrographs show tumour tissue sections stained with anti-caspase-3 antibody (original magnification, ×400) after NS, AAV-EGFP or AAV-PEDF treatment. (D) The caspase-3 stained index (%) of tumours treated with NS, AAV-EGFP, and AAV-PEDF (^*^P<0.05). (E) LLC tumours from NS, AAV-EGFP, and AAV-PEDF treated mice were stained with H&E (original magnification, ×400); noticeable necrosis was observed in tumours from AAV-PEDF treated mice.

More apoptotic cells in tumour tissue were observed in AAV-PEDF-treated animals than in AAV-EGFP- or NS-treated animals (Fig. 4A). The average percentage of apoptosis in the AAV-PEDF group was significantly increased compared with that in the AAV-EGFP and NS groups (Fig. 4B; P<0.05). Furthermore, there were more cell nuclei stained in the AAV-PEDF group compared with the other groups (Fig. 4C and D; P<0.05). As shown in Fig. 4E, histological analysis revealed that AAV-PEDF-treated tumours had increased cell death necrosis.

### AAV-PEDF inhibition of angiogenesis in vitro and in vivo

Endothelial cells form tube and capillary-like structures on a Matrigel membrane through a process involving attachment, alignment, and migration. Treatment with conditioned medium (CM) from LLC cells infected *in vitro* with AAV-PEDF significantly decreased this process (Fig. 3C). Quantitative analysis showed that AAV-PEDF restricted tube formation by 71 and 76%, respectively, in comparison to the AAV-EGFP or NS controls (Fig. 3D; P<0.01).

Alginate implants in AAV-PEDF-treated animals exhibited less vascularisation than AAV-EGFP- or NS-treated animals (Fig. 3E). We observed that the accumulation of FITC-dextran at the implant site in the AAV-PEDF group was significantly lower compared to the other groups (Fig. 3F, P<0.05).

The data confirmed that AAV-PEDF-mediated PEDF gene transfer and expression could suppress tumour angiogenesis in the studied tumour model.

### AAV toxicity in mice

**Figure 5:**
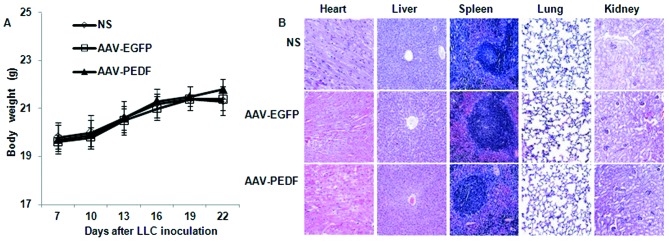
Changes in body weight and histological staining for basic organs. (A) There was no difference in body weight among the treatment groups. (P>0.1) (B) Basic organs from NS-, AAV-EGFP-, and AAV-PEDF-treated mice were stained with H&E (original magnification, ×200). There was no noticeable pathological change in tumours from AAV-EGFP- and AAV-PEDF-treated mice compared with NS control.

**Figure 6:**
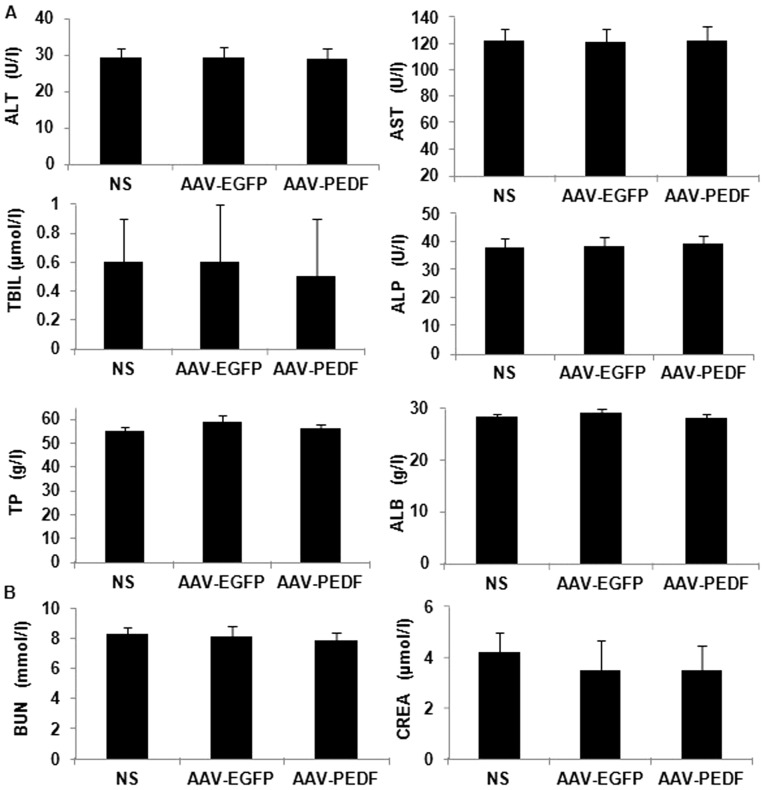
Mouse liver and kidney function. (A) There was no difference in mouse liver function of ALT, AST, TBIL, ALP, TP, and ALB in the respective treatment groups (P>0.1). (B) There was also no difference in the kidney function as measured by BUN and CREA (P>0.1).

We evaluated AAV toxicity in mice by monitoring changes in mouse weight, basic organ structure, and histological morphology, and analyzing mouse liver and kidney function. No significant difference was observed in body weight among AAV-EGFP-, AAV-PEDF- or NS-treated mice (Fig. 5A, P>0.1). Liver and kidney function data are shown in Fig. 6 (P>0.1). In addition, no apparent pathological changes were observed in the heart, liver, spleen, lung, and kidney tissue from the different groups as indicated by H&E staining (Fig. 5B).

## Discussion

Angiogenesis, the complex biological process by which new blood vessels develop from pre-existing ones, is known to play an essential role in supporting progressive tumour growth (1–3). Therefore, targeting neoangiogenesis or signals that promote neovessel growth is a promising anticancer therapeutic strategy (4–6). However, neovasculature growth is controlled by maintaining a balance between pro- and anti-angiogenic factors (7,8). For this reason, overexpression of anti-angiogenesis factors could be excellent therapeutic tools in combating tumour angiogenesis.

Combating lung cancer remains a major clinical challenge. Existing therapeutic protocols are very disappointing. Previous studies have demonstrated the efficacy of anti-angiogenesis therapy (4–6). In this study, we demonstrated that overexpression of PEDF mediated by the AAV vector exerts a remarkable suppression of tumour growth and prolongs animal survival in a C57BL/6 mouse model. The tumours treated with a single intratumoural injection of AAV-PEDF began to grow more slowly than the other two groups on Day 6 after treatment with maximum tumour growth inhibition observed on Day 15 after treatment (56% and 58% inhibition, respectively, compared with AAV-EGFP- or NS-treated mice). Tumour growth suppression was related to decreased microvessel density and increased apoptosis in AAV-PEDF-treated tumours. A 73% decreased MVD in the AAV-PEDF-treated tumours (Fig. 3A and B) closely paralleled the 58% reduction in tumour size, implying a direct relationship between lung carcinoma vascularity and growth. The antiangiogenic activity of PEDF was further demonstrated by tube formation and the alginate-encapsulated tumour cell assay (Fig. 3C-F). The average percentage of apoptosis in the AAV-PEDF group (10%) was significantly increased, compared with the AAV-EGFP (3%) and NS (3%) groups (Fig. 4B; P<0.05). These observations suggest that hindering the vascular supply to a tumour significantly curbs its ability to grow and that restriction of tumour angiogenesis caused an increase in tumour cell apoptosis in AAV-PEDF treated mice.

The mechanisms by which PEDF reduces neovascularisation are still largely unknown. Nevertheless, 2 major pathways have been implicated, namely, endothelial cell apoptosis via activation of the Fas/Fas-L death pathway (28) and disruption of the crucial balance between pro- and anti-angiogenic factors, in particular downregulation of VEGF expression (29–31). Whereas pro-angiogenic factors stimulate endothelial cells that express Fas, PEDF increases FasL expression on the endothelial cell surface. A caspase-dependent apoptotic cascade is subsequently initiated, resulting in endothelial cell death. Another mechanism underlying the antiangiogenic property of PEDF is that it also has an inhibitory effect on VEGF-induced angiogenesis. A study by Cai *et al* reported that PEDF activates γ-secretase-dependent cleavage of the VEGF-R1 C terminus, which consequently modulates angiogenic signalling via VEGF-R2 (31). Exogenous PEDF downregulated VEGF expression at both the mRNA and protein levels in the MG63 osteosarcoma cell line (26).

In this present study, our data indicated that PEDF not only acts to halt angiogenesis, but also has the ability to increase apoptosis in tumours. However, the mechanisms of PEDF-mediated apoptosis in tumours are still poorly understood. The apoptotic activity is likely due to a distinct functional epitope on the PEDF protein that was discovered by Filleur *et al*, who reported increases in prostate cancer cell death *in vitro* with the 34-mer peptide, but not with the 44-mer (20). Significant tumour cell apoptosis with full-length PEDF treatment *in vitro* has been observes in melanoma and osteosarcoma cells (16,26). This effect may be mediated by the Fas/Fas-L cascade similar to endothelial cell apoptosis, as it was reversed by neutralizing antibodies against Fas-L (16,26).

AAV has several excellent advantages making it a particularly promising vector for gene therapy. AAV has long-term transgene expression in experimental gene therapy methods compared with other viral vectors (37). More importantly, AAV is a non-pathogenic virus and has low immunogenicity because the genes encoding the wild-type protein are absent, and it has a replication-defective nature (32–34). In our study, we did not detect AAV-induced toxicity as measured by calculating the change in animal body weight, observing basic organ structure and histological morphology, and analyzing mouse liver and kidney function (Figs. 5 and 6). Several recent reports have demonstrated the therapeutic efficiency of PEDF viral vector intratumoural injections (20,38,39). Intratumoural gene therapy allows for selective expression within tumour cells contributing to higher intratumoural concentrations while maintaining normal systemic levels, thus limiting the number of adverse reactions. AAV vectors in gene therapy could be relatively safe and efficient.

In conclusion, our results indicate for the first time that AAV-PEDF by intratumoural injection can provide an effective protocol for LLC treatment in a C57BL/6 mouse model. Mechanisms of PEDF-mediated reduction of LLC tumour growth include both inhibition of tumour angiogenesis and stimulation of tumour cell apoptosis.

## Abbreviations

PEDF: pigment epithelium-derived factor
AAV: adeno-associated virus
MVD: microvessel density
LLC: Lewis lung carcinoma
VEGF: vascular endothelial growth factor
HUVECs: human umbilical vein endothelial cells
NS: normal saline
VEGF-R1: vascular endothelial growth factor-receptor 1
VEGF-R2: vascular endothelial growth factor-receptor 2
